# An International Dental Congress

**Published:** 1887-02

**Authors:** 


### Editorial, Etc.

---

AN INTERNATIONAL DENTAL CONGRESS.—A conference of dentists who were in attendance at the Eighteenth Anniversary of the First District Society of New York, was held in New York City, to consider the feasibility and advisability of taking steps for the calling of an International Dental Congress at some future date. Some thirty-five dentists were in attendance.

The meeting was called to order, and elected Dr. W. H. Dwinelle chairman, and Dr. George A. Mills secretary.

Dr. Kingsley opened the discussion by some remarks in which he considered the advisability of taking steps for organizing a meeting to be called an International Dental Congress, and he offered the following resolution:

*Resolved.* That in the opinion of the conference the interests of the dental profession throughout the world will be advanced by an International Dental Congress.

A discussion then followed, in which Drs. Northrop, Truman, Brophy, Hunt, Winder, Keech, Kingsley and the chairman took part. There was no difference of opinion as to the advisability of holding such a congress at some future time, and the years 1888, 1889 and 1891 were severally suggested and considered. The year 1890 was out of the question, because of another International Medical Congress to be held in that year. As to the time, no decision was reached. The resolution was carried unanimously. A second resolution was offered, viz.,

*Resolved.* That a committee of temporary organization be appointed, whose duty it shall be to make such a plan for a permanent organization as shall in their estimation best call out universal support.

This was also discussed and carried.

A third resolution was offered and carried, viz.,

*Resolved.* That this committee be empowered to fill vacancies and enlarge its numbers at their discretion.

This conference was amicable in a large sense, yet there was a free interchange of opinion. While all did not think alike in all things, wise measures were strongly advocated, so that it should not appear that there was any disposition to place obstructions in the way of any movement that sought the best good of all.

The meeting adjourned subject to the call of the chair.

